# Cell labeling with magnetic nanoparticles: Opportunity for magnetic cell imaging and cell manipulation

**DOI:** 10.1186/1477-3155-11-S1-S7

**Published:** 2013-12-10

**Authors:** Jelena Kolosnjaj-Tabi, Claire Wilhelm, Olivier Clément, Florence Gazeau

**Affiliations:** 1 Laboratoire Matière et Systèmes Complexes, UMR 7057, CNRS and Université Paris Diderot, France; 2 Inserm U970, Paris Cardiovascular Research Center-PARCC/Université Paris-Descartes, France

**Keywords:** High-resolution magnetic resonance imaging (MRI), cellular MRI, magnetic nanoparticles, magnetic cell labeling, magnetic vectorization

## Abstract

This tutorial describes a method of controlled cell labeling with citrate-coated ultra small superparamagnetic iron oxide nanoparticles. This method may provide basically all kinds of cells with sufficient magnetization to allow cell detection by high-resolution magnetic resonance imaging (MRI) and to enable potential magnetic manipulation. In order to efficiently exploit labeled cells, quantify the magnetic load and deliver or follow-up magnetic cells, we herein describe the main requirements that should be applied during the labeling procedure. Moreover we present some recommendations for cell detection and quantification by MRI and detail magnetic guiding on some real-case studies *in vitro *and *in vivo*.

## Rationale

Magnetic labeling provides living cells with new features, which allow cell magnetic resonance imaging (MRI), enable distal cell manipulation applicable to tissue-engineering techniques, or could be even used for magnetically assisted cell delivery to target organs *in vivo*. Among magnetic nanoparticles, superparamagnetic iron oxide nanoparticles have an extensively documented background about particle synthesis and surface modification. Moreover, if properly used (i.e. when well dispersed), such particles do not alter viability, function, proliferation or differentiation of cells. In order to efficiently and safely label different cell types, including stem cells, this tutorial presents a well-established method of controlled cell labeling with citrate-coated ultra small superparamagnetic iron oxide nanoparticles (herein referred to as magnetic nanoparticles - MNP). In addition, we also provide a method of detection and quantification of single cells with high resolution MRI and describe the basis of cell sorting and magnetic manipulation for engineering and therapeutic purposes.

## Cell labeling with magnetic nanoparticles

### Background

Different strategies can be applied in order to endow cells with sufficient magnetization to be detectable by MRI and/or to be manipulated by an external magnetic field. The handiest way is the co-incubation of cells with magnetic nanoparticles, where the particles are generally internalized through the spontaneous endocytosis pathway [1] or phagocytosis [2]. However cellular uptake may strongly depend on nanoparticle properties, especially on surface functionalization [3]. While dextran-coated nanoparticles show very poor uptake due to steric repulsions between particles and cell membrane, the best strategy to facilitate endocytosis of nanoparticles is to favor a specific binding or non-specific adsorption to the cell membrane. This can be achieved by linking biological effectors on nanoparticles such as antibodies, transferrin or HIV-Tat peptide that target specific receptors on plasma membrane [4]. The use of cationic transfection agents that form highly charged complexes with nanoparticles is also efficient to trigger cellular uptake, but usually requires long incubation times (>6 hours) [5]. Moreover the aggregation state of nanoparticles in the formed complexes cannot be controlled.

### The importance of nanoparticle stability in cell labeling medium

As the cells react in a different manner depending on whether the nanoparticles remain dispersed in suspension or become aggregated, the stability of MNPs is a key issue to achieve an efficient and controllable magnetic labeling. Moreover, cell toxicity might arise from MNPs aggregates, whereas the same MNPs would have no deleterious effect when correctly dispersed. In addition, the surface properties of nanoparticles can be changed upon dynamic adsorption of the proteins and macromolecules encountered in the biological medium. Therefore what the cell perceives is not the original nanoparticle designed by a chemist, but a modified heterogeneous surface reconfigured by the biological milieu [6,7]. Both the physical state (aggregated versus isolated nanoparticles) and the biological identity of particles (comprising the adsorbed proteins) dictate the uptake by different cell types and the *in vivo *biodistribution of nanoparticles.

### Practical aspects of cell labeling

**Figure 1:**
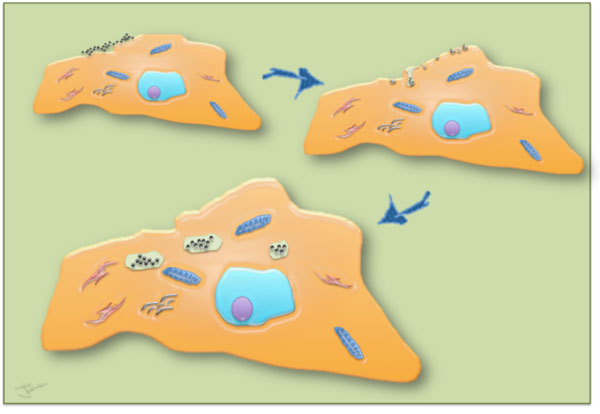
**Schematic representation of cell interactions with nanoparticles**. Particles first adsorb on plasma membrane, which consequently undergoes invagination. The MNP-loaded vesicles then pinch off the membrane and subsequently fuse with endosomes and lysosomes, which are dispersed within the cell's cytosol.

**Figure 2:**
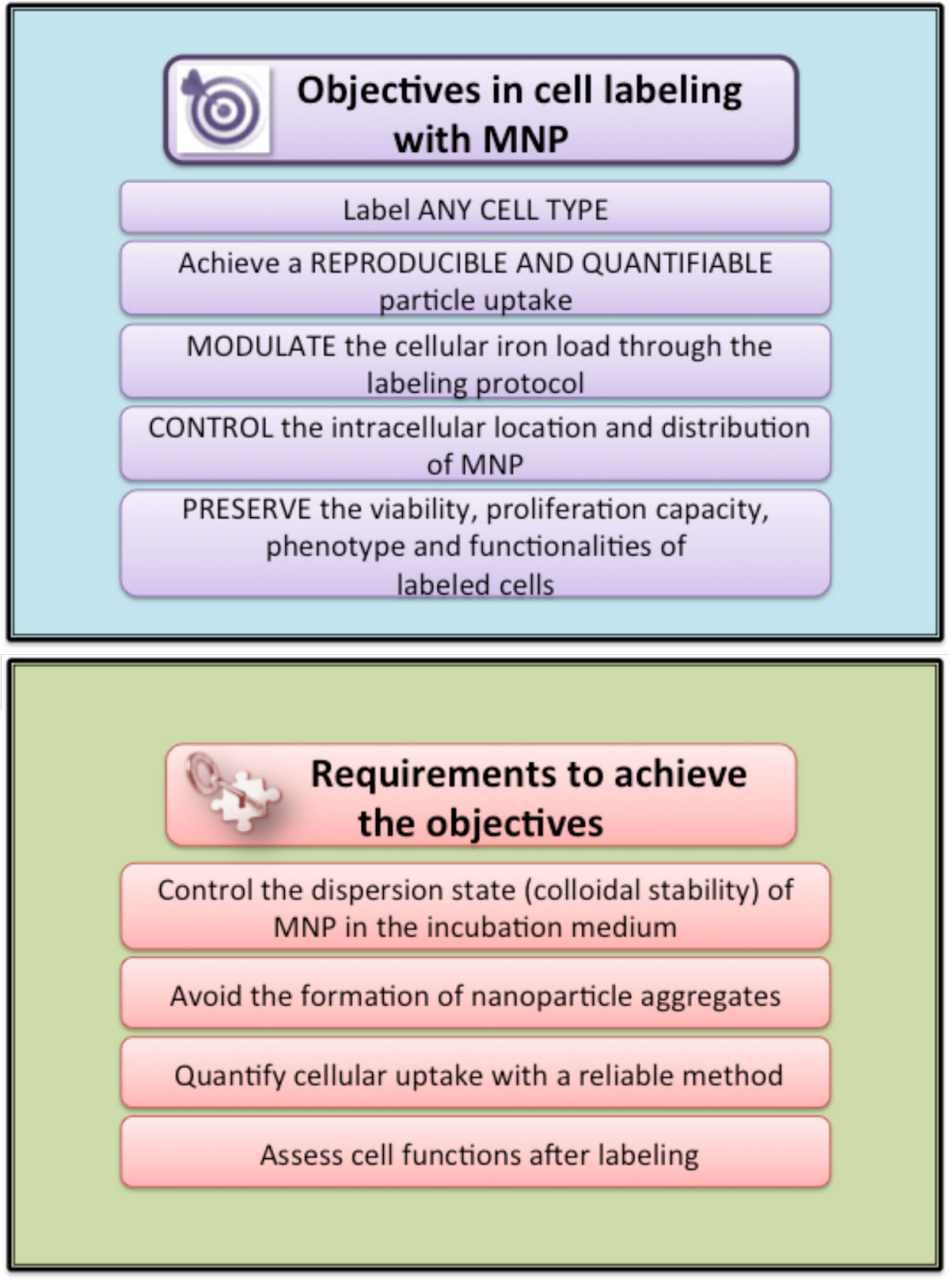
**Schematic representation of the objectives and key requirements for efficient cell labeling**.

**Figure 3:**
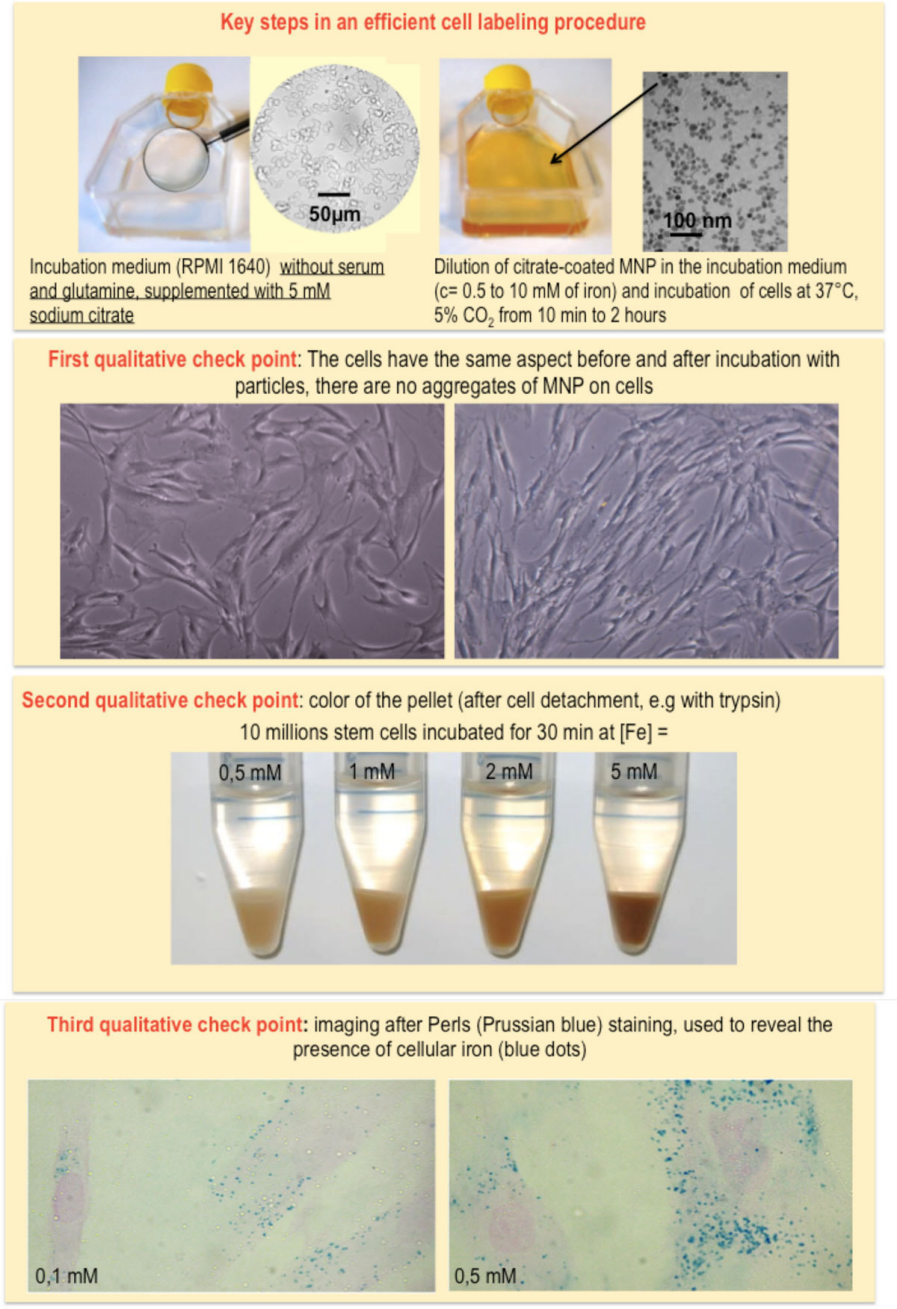
**Labeling procedure and its qualitative checkpoints to assess labeling efficacy**. The figure represents the key steps for efficient cell labeling. The checkpoints include the evaluation of cell outlook (color, shape, presence of aggregates).

**Figure 4:**
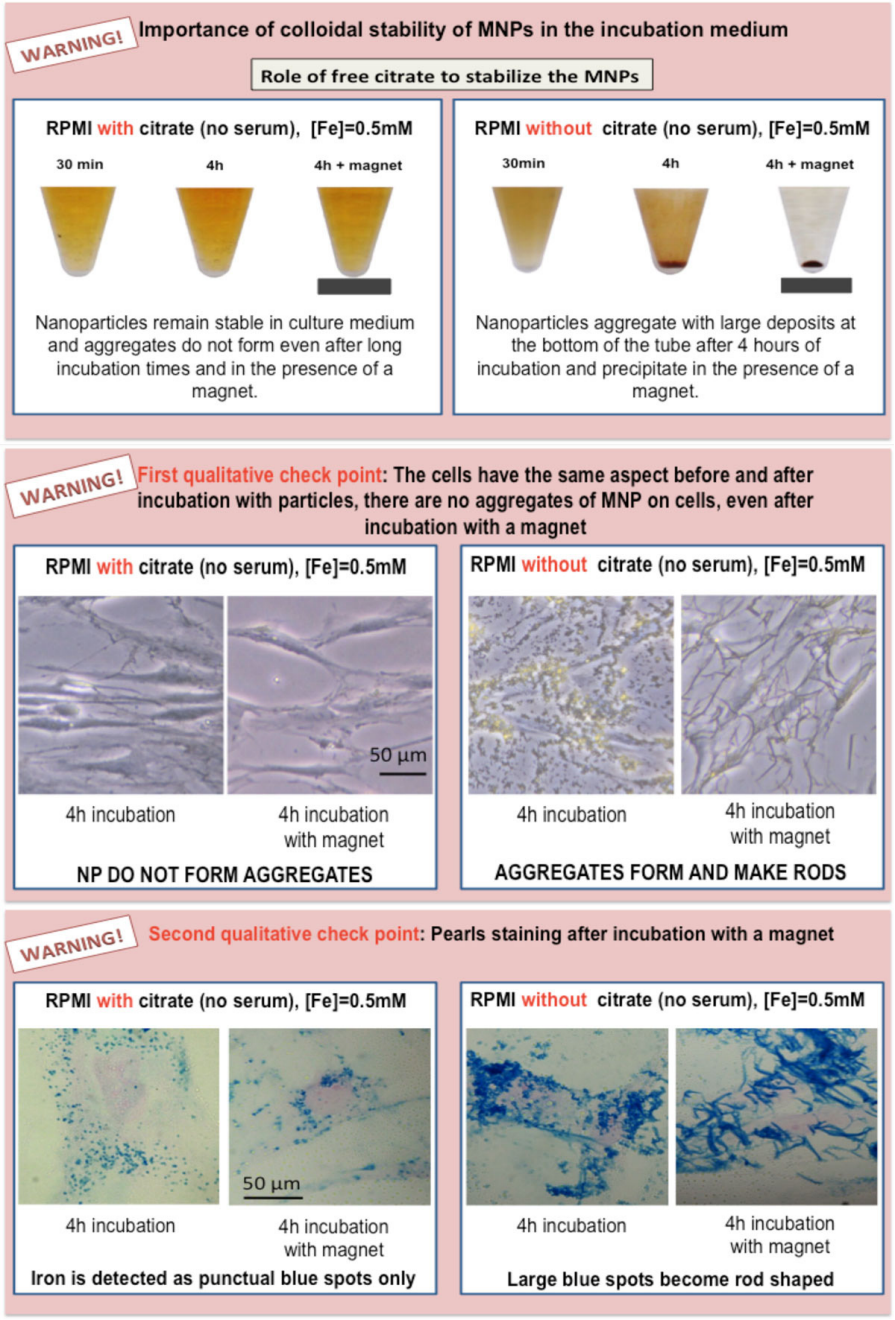
**Comparison of examples of appropriate and inappropriate labeling conditions due to aggregation of anionic magnetic nanoparticles**.

Labeling cells *in vitro *offers the opportunity of controlling cell interactions with nanoparticles (Figure 1). In this tutorial we describe a simple and straightforward method to magnetically label virtually all cell types in a rapid, predictive and quantitative way. The objectives and requirements for an efficient cell labeling are summarized in Figure 2 and the key steps in the labeling procedure are shown on Figure 3. Our method uses citrate-coated maghemite nanoparticles of 7-8 nm in diameter. Small citrate ligands on the surface of the iron oxide confer negative surface charges to the particles, which are stabilized by electrostatic repulsions in water or serum-free culture medium. We use serum-free culture medium to avoid adsorption of proteins on the nanoparticles that could affect both their stability and their affinity for the cell membrane. Moreover the stability of citrate-coated particles (measured through their hydrodynamic size) can be modulated by controlling the concentration of free citrate ions: the nanoparticles remain isolated in culture medium supplemented with 5 mM free citrate, while they aggregate in citrate-free medium and eventually form chains when submitted to a magnetic field [8] (Figure 4). Thus, to avoid MNP aggregation, cell labeling should be performed in the serum-free medium supplemented with citrate. While appropriate labeling conditions preserve the homeostasis of cells, failure to provide optimal labeling conditions might lead to particle aggregation (Figure 4) that might have a negative impact on the cells.

After a short incubation time (typically less than one hour, compared to several hours of cell labeling with other types of magnetic nanoparticles), cells are rinsed with the citrate-enriched, serum-free medium and left for particle chase in the standard cell medium at 37°C. Once the chase period is over, cells appearance should be attentively examined. The main qualitative check points are summarized in Figure 3.

### Mechanistic aspects in cell labeling with MNPs

**Figure 5:**
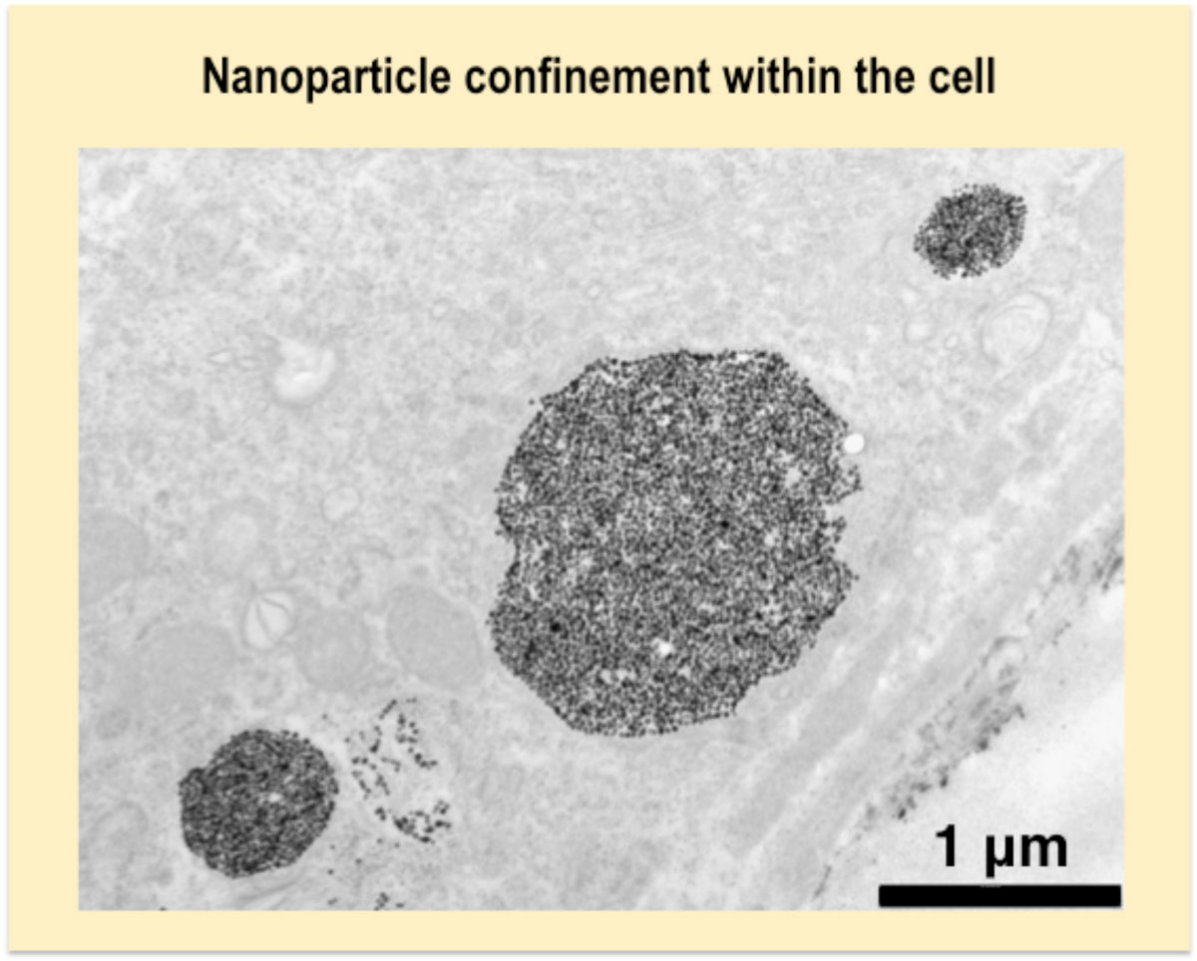
**Transmission electron micrograph of a cell loaded with magnetic nanoparticles, which are confined in endosomes or lysosomes**.

The uptake of dispersed citrate-coated MNPs consists of a two-step process. The first step is the non-specific adsorption of particles on the plasma membrane, following a generic Langmuir kinetics. This step can be investigated separately if cells are maintained at 4°C, thus inhibiting the internalization process. Remarkably the affinity of MNPs for cell membrane does not depend on cell type (1.6-4 × 10^7 ^M^-1^) and the binding capacity (typically 0.03 pg/µm^2 ^or 2.4 × 10^4 ^nanoparticles/µm^2^), but it only depends on cell size [9]: the larger the cell, the higher the number of nanoparticles adsorbed on plasma membrane. The second step involves the internalization of the plasma membrane, which invaginates, encloses the nanoparticles into vesicles, and delivers them into intracellular compartments, successively to early endosomes, late endosomes and ultimately to lysosomes (Figure 5). At 37°C both the particles adsorption and internalization occur concomitantly. Moreover, the binding sites on plasma membrane are continuously recycled, allowing continuous internalization. As the internalization capacity and the internalization time are conserved for different cell types (with the exception of macrophages), this model allows predicting quantitatively the cellular uptake and optimizing the labeling procedure in terms of incubation time and extracellular iron concentration.

### Intracellular storage of internalized particles

Intralysosomal sequestration of MNPs has the advantage to protect the cell from the release of any free toxic iron species in the cytoplasm. Moreover the lysosomes are used by cells to metabolize MNPs and to degrade them at long term [10,11]. Likewise the *in vivo *biotransformation of MNPs occurs intracellularly within the lysosomes, and the iron, coming from the degradation of MNPs, is locally transferred and stored within the ferritin, the iron storage protein [12,13].

### Impact of magnetic nanoparticles on cell viability

**Figure 6:**
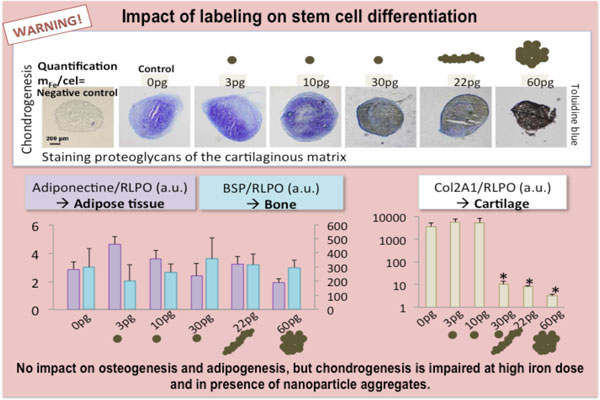
**Example of monitoring of cell functions after mesenchymal stem cell labeling**. The figure is adapted from reference (8) and shows cell differentiation ability. In the represented case, after labeling, cell differentiation to adipose or bone cells is not impaired at high MNPs concentration. In contrast, high MNP load impacts cartilage formation.

Indeed, one of the significant aspects in cell labeling is also the assessment of cell functions after MNP internalization. Prior to the use of magnetically labeled cells for imaging and therapeutic purposes, functional tests have to be performed in order to check the innocuousness of magnetic labeling on cell viability, cell proliferation, cell phenotype and specific functionalities. Cell viability can be assessed by different assays, which may determine different cell characteristics, such as the integrity of cell membrane, mitochondrial activity, apoptosis, etc. While there are no special recommendations on which tests to use after MNP labeling, we should compare results from the same kind of assay if we are comparing viabilities of different cells. Cell proliferation should be monitored over a period of at least 5 days [9]. The preservation of cell functions and differentiation capacities might differ among distinct cell types, therefore should be determined specifically. The expression of specific genes of interest can also be quantified to assess subtle phenotypical alterations following SPIO labeling [14,15]. An example is given in Figure 6[8].

To date different cell types have been labeled with MNPs (immune cells, endothelial cells, cancer cells, primary culture or established cell lines and progenitors cells, to mention just a few) and detrimental effects on cell proliferation and cell functions at short and long terms, *in vitro *or *in vivo*, were not observed [9]. The labeling of stem cells is more tricky as these cells should conserve their self-renewal and multipotency after internalization of MNPs [16]. Human neural precursor cells were also efficiently labeled without impairment of their differentiation capacity [17,18]. However in some studies using transfection agents for cell labeling, controversial effects were observed on the multilineage differentiation capacity of mesenchymal stem cells. The chondrogenesis (i.e. the capacity to differentiate in cells of cartilage) was partially inhibited in one study [19], but not in others [14,20-22], whereas adipogenesis and osteogenesis were not impaired. On the contrary, while labeling cells with citrate-coated MNP, we could modulate the amount and the physical state of nanoparticles interacting with cells and could conclude that only high dose of MNPs or an aggregated state, could have adverse effects on cell differentiation (chondrogenesis) [8] (Figure 6). Labeling conditions with perfectly stable MNP is thus recommended for use in cell therapy assays.

### Fate of the particles in a living cell

During the division process, the cell shares the magnetic endosomes between its two daughter cells. The iron load is thus reduced by a factor of two at each division. In normal conditions, there is no exocytosis of MNPs. However, under stress conditions, some magnetically labeled cells can release nanoparticle-loaded microvesicles in the extracellular medium [23,24]. These cell-released vesicles can transfer nanoparticles to other naïve cells [24], especially macrophages [25]. This process, if confirmed *in vivo*, could participate to a horizontal intercellular transfer of nanoparticles, challenging to some extent the initial specificity of cell labeling [26,27].

### Quantification of iron load

**Figure 7:**
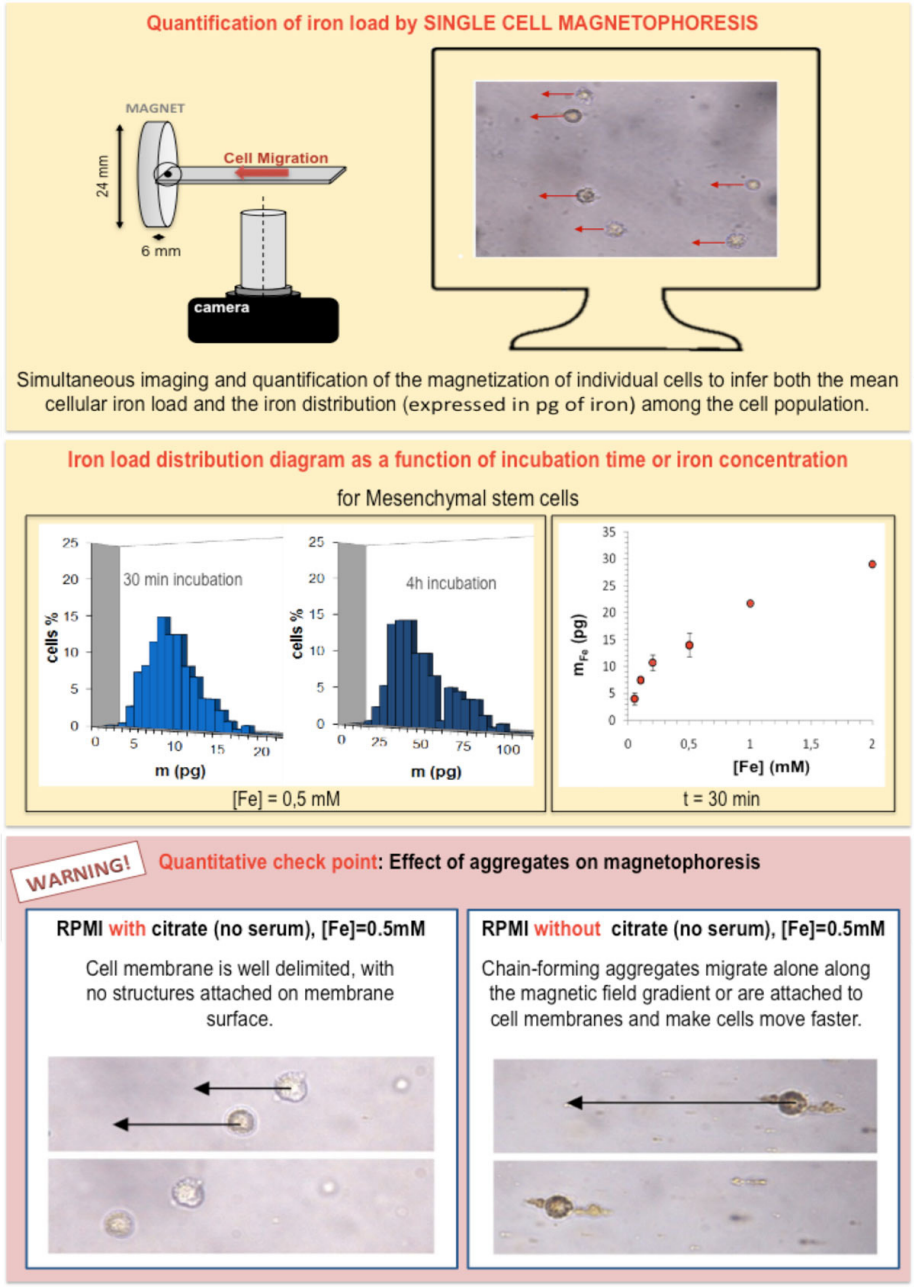
**Single cell magnetophoresis**. Schematic representation of the magnetophoresis setting (top) and iron load distribution diagrams (middle) obtained by the magnetophoresis experiment, presented as iron load as function of time or as function of iron concentration in the cell culture medium. When magnetophoresis is performed on cells that have not been correctly labeled, the outcome of the assay does not reflect the correct value of the intracellular iron load (as the obtained value is higher due to extracellular aggregate pods).

Once the chase period is over, cells appearance should be attentively examined. Check points are summarized in Figure 3. After our first qualitative examinations (Figure 3), we should proceed with the quantification of iron load. Currently there are several methods for iron dosage in cells, namely, the elemental analysis, the electron paramagnetic resonance (a method which relies on magnetism and allows the differentiation between superparamagnetic iron from the particles and endogenous iron bound in ferritins, the iron storing proteins) [12,28] and the colorimetric analysis, to mention just a few. Apart from these methods that are generally applied to the cell pellet, other single cell iron assessments can be also performed. Something of the kind is the single cell magnetophoresis [28]. This method also relies on magnetism and is schematized in Figure 7.

### Cell responsiveness to the magnetic forces

As lysosomes in labeled cells concentrate several millions of MNPs, a labeled cell becomes responsive to an inhomogeneous magnetic field, generated, for example, by a permanent magnet. In a non uniform magnetic field B, defined by an unidirectional magnetic field gradient gradB, a labeled cell experiences a magnetic force M(B)gradB, where M(B) is the magnetic moment of the cell in the field B (equal to the magnetic moment of one MNP multiplied by the number of MNPs per cell). Typically a permanent magnet generates a magnetic field gradient of 10-50 T/m over a distance of approximately 1 cm. The corresponding force experienced by the cell (with an average iron load of 10 pg) may vary from 1 pN to a few nN [29]. For cells in suspension, the magnetic force is balanced by the viscous force 6πηRV, where η is the viscosity of the medium, R the cell radius and V the cell velocity. In a set-up with calibrated B and gradB (18 T/m), it is easy to deduce iron load from the determination of V and R for each cell by video-microscopy (Figure 7 top). From this experiment we can thus determine the distribution of MNP uptake in a cell population (Figure 7 middle). If the cells have not been labeled in the appropriate way (and are consequently covered with particle aggregates and cellular debris), magnetophoresis will not reflect the cell velocity that is due to intracellular iron, but will indicate the velocity that is due to internalized *and *membrane-attached nanoparticles. Besides, as we can see on Figure 7 (bottom), chains of aggregates that are not attached to cell membranes also migrate towards the magnet. In contrast to other global dosage of iron load in cell pellet, single cell magnetophoresis allows to visualize potential artifact linked to nanoparticle aggregation. The control of nanoparticle stability is once again the critical point to achieve a quantifiable and reproducible magnetic labeling.

## Imaging cells with magnetic resonance imaging (MRI)

### Cell tracking *in vivo*: the advantages of MRI

One of the new emerging applications of magnetic cell labeling concerns magnetic resonance cell tracking. Magnetic resonance imaging (MRI) allows real-time whole-body examinations with excellent soft-tissue contrast and spatial resolution. Moreover, impactful development has been made on high-field MR scanners, magnetic gradient systems and radiofrequency (RF) coils [30]. One of the new coils, such as the cryogenic probe, allows sub-milimetric resolution and gives the means to perform cellular MRI *in vivo*. The advantage of the cryogenic probe to improve the signal-to-noise (SNR) ratio and concomitantly improve the image resolution, has been demonstrated throughout the last decade in several studies [30,31].

### Iron oxide nanoparticles as cellular MRI contrast agents

**Figure 8:**
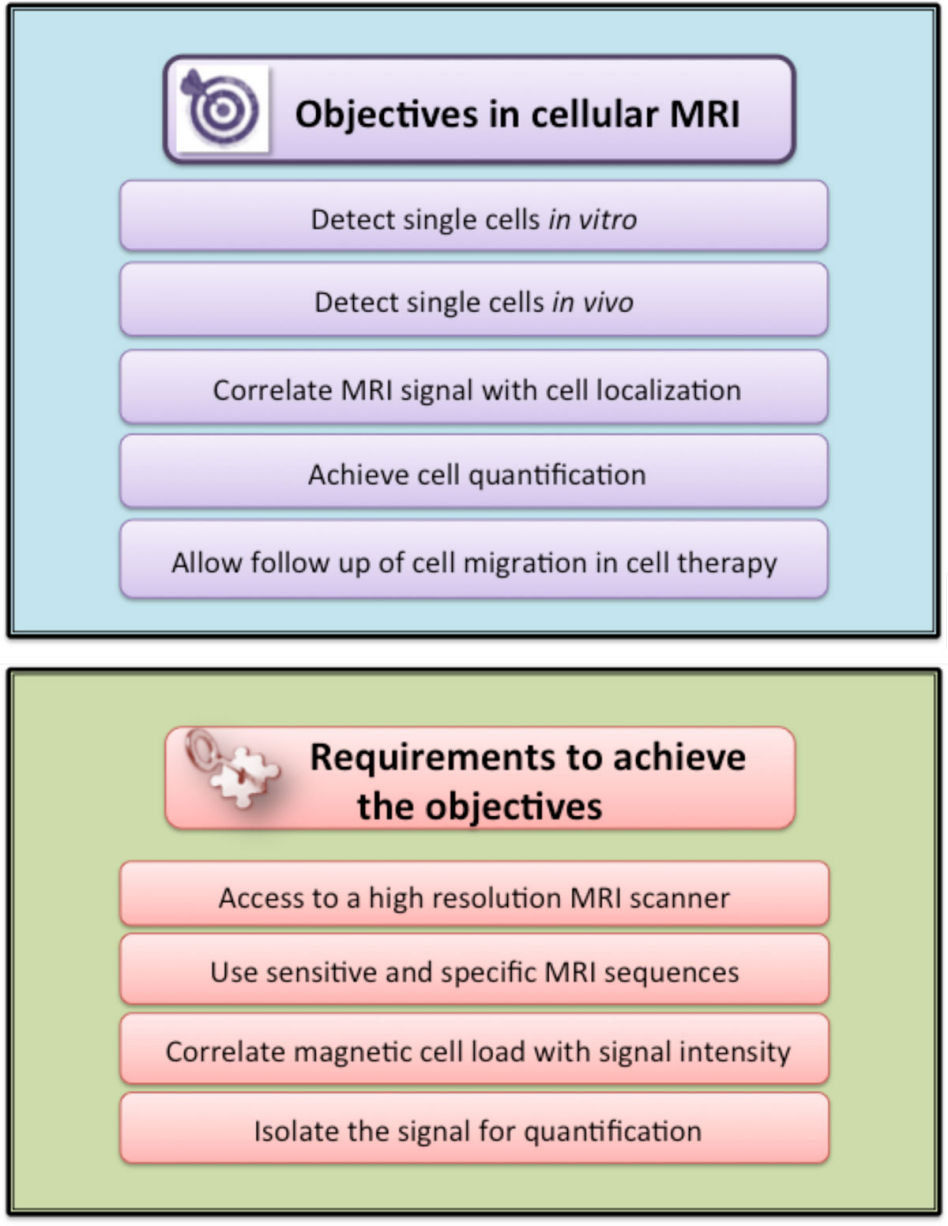
**Schematic representation of the objectives and key requirements for efficient cell imaging by MRI**.

**Figure 9:**
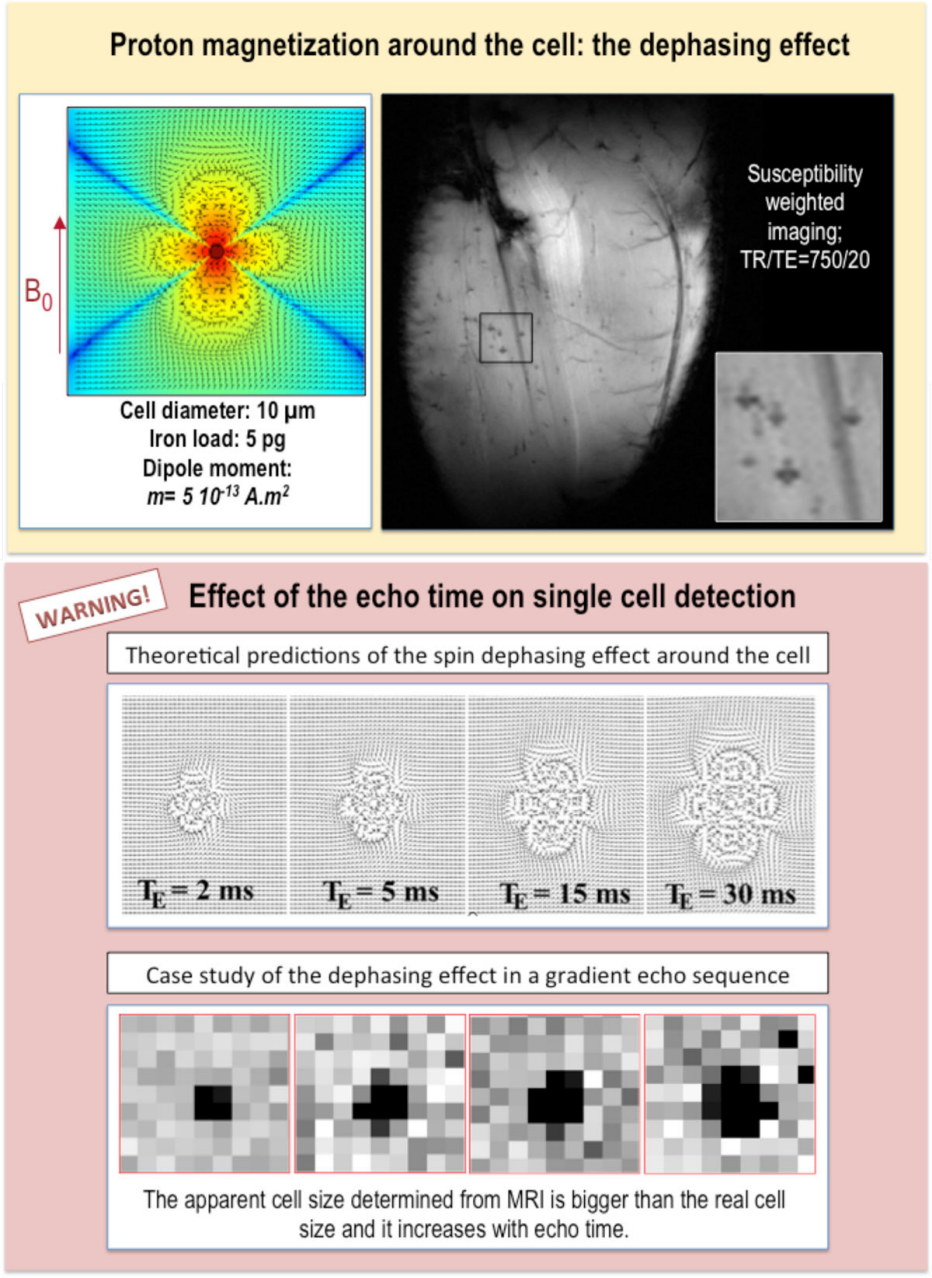
**The MR dephasing effect**. Theoretical and real case study of the dephasing effect of protons in the vicinity of a labeled cell *in vivo*. The upper panel shows the MR image of the lower hind limb of a mouse, intravenously injected with magnetically labeled macrophages, which form a typical four-lobed clover in the susceptibility-weighted scan (in-plane resolution of 39 μm), obtained with a 4.7 T scanner provided with a dedicated cryogenic probe. The bottom panel points out the impact of the echo time on the apparent cell size (top theoretical predictions and bottom real case study obtained at 9.4 T). The bottom panel has been adapted from reference (46).

**Figure 10:**
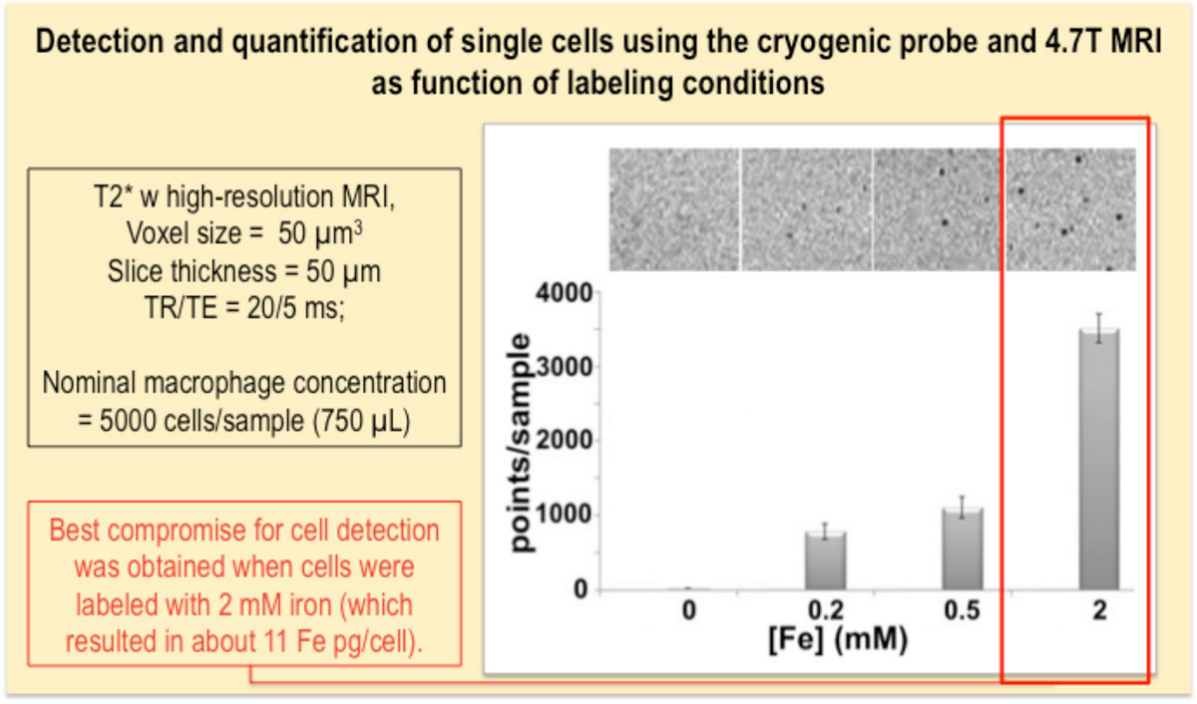
**Cell detection as function of labeling conditions**. A case study of agarose phantoms spiked with the same amount of cells, labeled with ascending concentrations of iron. The images were obtained with a T2* weighted gradient echo sequence, with a 4.7 T scanner provided with a dedicated cryogenic probe. The figure is adapted from reference (31).

In order to be distinguished from tissues, the cells have to be labeled with a contrast agent (Figure 8). Iron oxide nanoparticles are potent MR contrast agents that can positively or negatively enhance the signal, depending on particle concentrations and applied MR sequence [32]. The particles are characterized by their r1 and r2 relaxivities, which indicate the ability to increase the longitudinal and transverse relaxation rate of proton magnetization per mM of agent. Nevertheless, when MNPs are internalized into endosomes or lysosomes, their contrast properties radically change [33]: their longitudinal relaxivity is strongly diminished due to poor accessibility of water protons among highly concentrated nanoparticles tightly packed in endolysosomal compartments. Once within the intracellular compartments, magnetic interactions between MNPs also likely play a role in relaxivity variations, increasing the r2/r1 ratio after cell internalization [34]. An important consequence is that a magnetized cell creates a strong localized magnetic inhomogeneity in the uniform magnetic field of the MR scanner. Typically the field increment is about 10^-4 ^T at the surface of a cell (loaded with approximately 5 pg of iron) and falls to 10^-7 ^T at a distance of 50 µm from the cell [33]. This cell-induced magnetic artifact (Figure 9) can be detected with susceptibility weighted imaging or T2* weighted gradient echo sequences. Consequently, when spatial resolution is sufficiently high (typically less than 100 µm), single magnetic cells appear as focal signal voids. Single cell detection has been proven using high field MRI [33] or the clinical 1.5 T scanner equipped with a low noise superconducting coil [35]. As an example, Figure 10 shows high-resolution MR scans of agarose gels containing different numbers of individualized magnetically labeled cells, obtained at 4.7 T with a scanner provided with a cryogenic probe [31]. At low density, each signal void can be associated to one single cell. Indeed, the apparent cell size, detected by MR, is larger than the actual size of the cell and depends on the parameters of the MR sequence (Figure 9).

### Cell imaging in cell therapies

**Figure 11:**
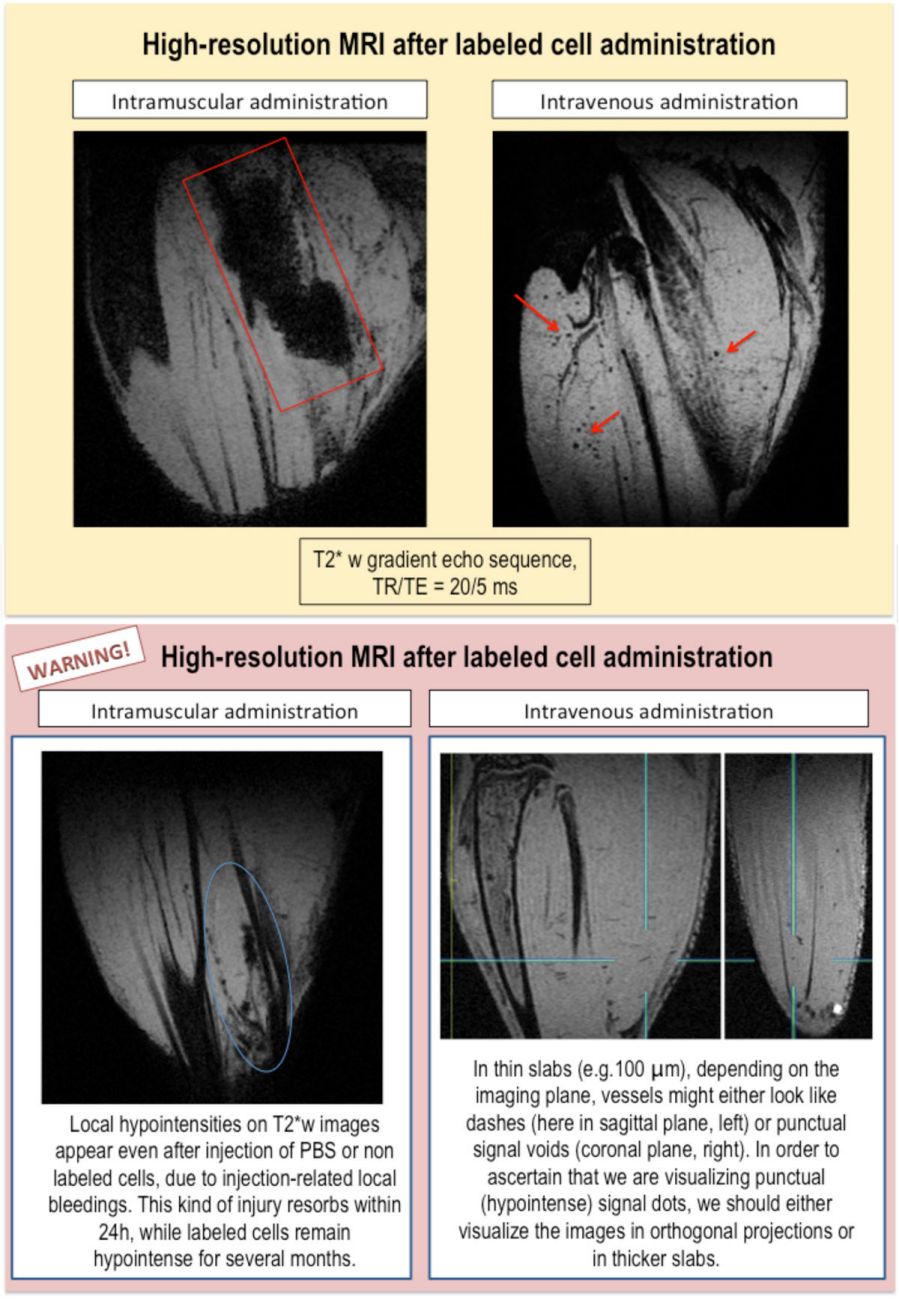
**High-resolution of murine hind limbs injected with labeled cells or phosphate saline buffer (PBS) only**. Top: mice intra-muscularly (left) or intravenously (right) injected with labeled macrophages. Bottom: mice intra-muscularly (left) or intravenously (right) injected with PBS. The images were obtained with a 4.7 T scanner provided with a dedicated cryogenic probe.

Cell tracking by MRI has become a method of choice to evaluate cell therapies, which involve direct (local or intravenous) administration of labeled cells (Figure 11). At high cell densities (local injections), isolated cells can be hardly detected, but we can observe a global signal loss, which is less dependent on MRI parameters (Figure 11). In a pioneering study using cellular MRI *in vivo*, MRI could be used to monitor the migration of lymphocytes injected intravenously to tumor bearing mice [36]. Lymphocytes were targeted to tumor cells through immune recognition, where the MRI showed a complex cell migration pathway. Lymphocytes first homed to the spleen to multiply and become activated, and only after multiplication they infiltrated the tumor and made it regress. This study was important from a methodological point of view, showing, for the first time, that single cells could be detected by MRI directly *in vivo *in a tumour [36]. This was extremely challenging, as lymphocytes were poorly labeled after several divisions in the spleen (and cell iron mass fell below 0.2 pg) and the imaging was made on a clinical 1.5T MR scanner. Together with many other studies by different groups, we can realize that MRI offers a great potential for cell tracking, which is also progressively being integrated in clinical assays. Be that as it may, despite the fact that MRI might provide us with real-time insight in cell distribution *in vivo*, we should corroborate its results with other, even *post-mortem *methods of cell detection, such as histology.

### Quantification of punctual signal voids

**Figure 12:**
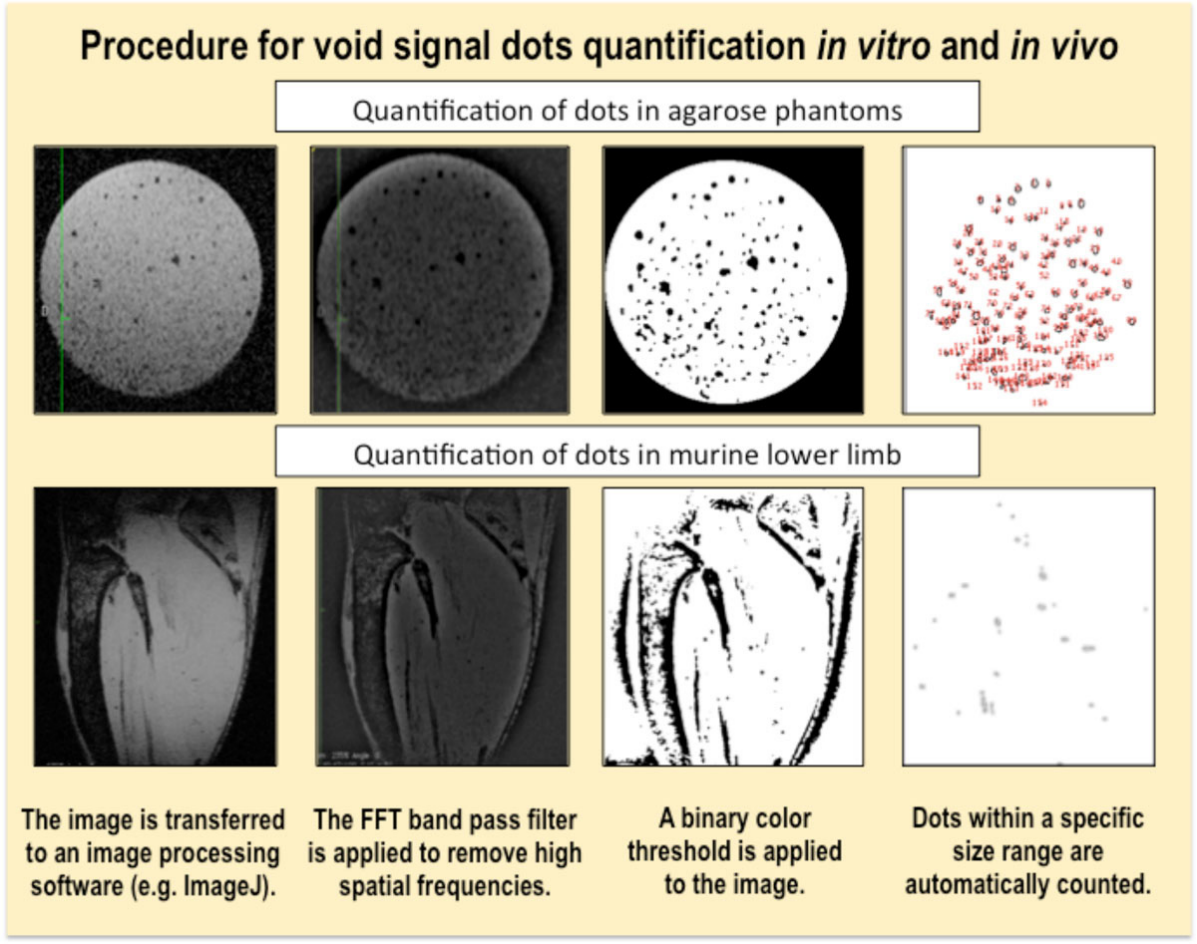
**Representation of a simplified procedure for relative dot quantification *in vitro *and *in vivo***. Image processing was performed with the open source ImageJ software.

In order to quantify detected signal voids that might correlate to administered magnetic cells, we should proceed with image processing and (automatic) dot count. In MR image processing, highly precise dot quantification remains very complex especially *in vivo *where several tissular structures might impact the dot count. However a good approximation can be made with ImageJ, the open source software from the National Institutes of Health. The step-by-step procedure for dot quantification obtained by ImageJ is illustrated on Figure 12.

## Magnetic manipulation of cells: from cell sorting to magnetic targeting in tissue engineering and cell therapies

### Magnetic cell sorting

**Figure 13:**
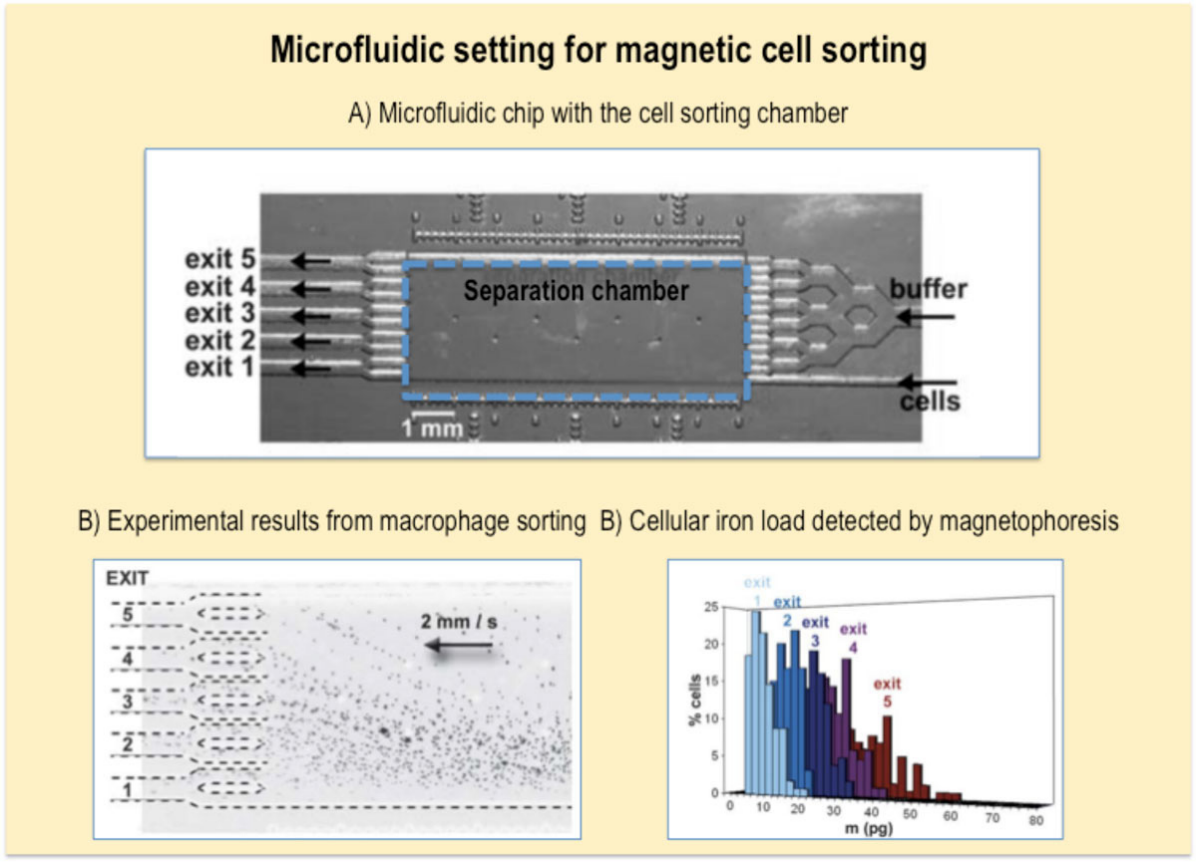
**Magnetic cell sorting set-up**. A) The photograph of the microfluidic chip showing the cell and buffer inlet, the separation chamber and five exit channels. Within the chamber, each cell population will move towards a specific exit. B) The migration of differently loaded macrophages towards their respective exits is driven by the value of the magnetic field gradient along the cell's trajectory and by the cell's magnetic load. C) The iron load of each cell fraction was quantified by the single-cell magnetophoresis. The figure is adapted from reference (38).

Magnetic cell manipulation that applies to magnetophoresis can also be applied to magnetic cell sorting (Figure 13), where we can separate cells in respect to their magnetic load [38]. This may be particularly advantageous when we want a precise and homogenous iron load within a cell fraction and/or want to eliminate poorly loaded cells that would, for example, be less detectable by MRI or less responsible to magnetic targeting. Moreover, magnetic cell sorting could be used to separate magnetic cells from complex mixtures or to sort cells with respect to their endocytosis capacity.

### Impact of the magnetic force

The effect of magnetic forces on cells will be also tightly related to the fact if the cell is suspended in a liquid or if it adheres on a substrate. While suspended cells more or less freely move when submitted to remote magnetic forces, when we try to magnetically manipulate adhering cells and the magnetic force is lower than the adhesion constraint, the cell cannot move and the magnetic force acts on MNP loaded intracellular endo-lysosomes. Such intracellular constraints can be used to deform the cell in a controlled direction and could be used, for example, to control the formation of a vascular network with magnetically labeled endothelial progenitor cells [39]. Magnetic manipulation might allow enhanced cell seeding and engraftment in different scaffolds for tissue engineering [40] and may enable new perspectives for *in vitro *construction of organized multicellular assemblies and tissue substitutes [41].

### Magnetic vectorization: the response to the need for localized cell delivery

**Figure 14:**
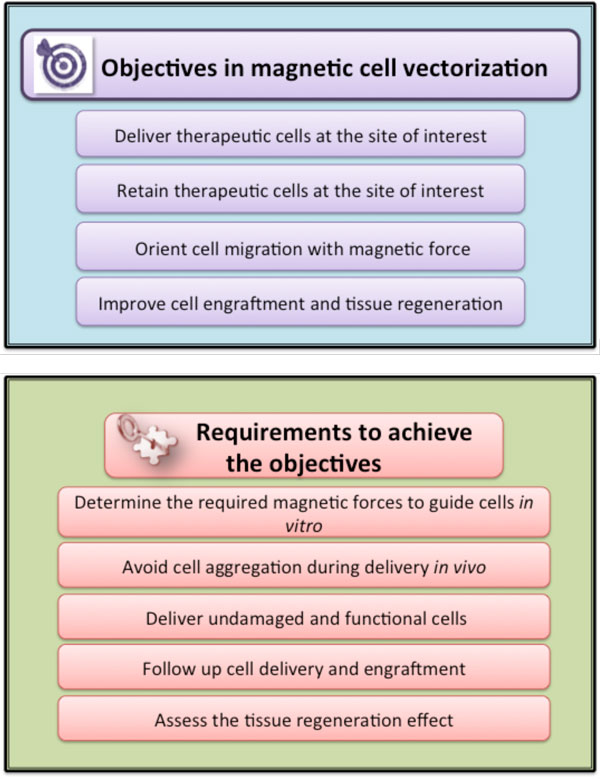
**Schematic representation of the objectives and key requirements for magnetic cell vectorization**.

For what concerns magnetic cell manipulation *in vivo*, a new field of therapeutic delivery- the magnetic vectorization or magnetic targeting of cells is emerging (see Figure 14 for the main objectives and requirements for magnetic cell targeting). This might be particularly interesting for cell guiding in stem cell transplantation for regeneration of injured tissues. Nevertheless while a substantial therapeutic effect was expected from stem cell treatment in acute or chronic cardiac ischemia, the treatment showed moderate therapeutic benefit in preclinical trials. Stem cell transplantation failed to improve the myocardial function, mainly because the majority of injected cells escape from the injured site, due to the local blood flow, which washes away the cells, and cardiac contractions, which squeeze the cells out, leading to poor cell engraftment (generally only less than 10% of the originally injected cells remain in the injured area). In order to enhance cell retention, some recent studies have proposed magnetic targeting in different cell therapies. However, to date, it is still uncertain if magnetic forces applied to cells would overcome the forces induced by the bloodstream and/or if the cells would still persist in the target place, once the magnet would be withdrawn.

Magnetic vectorization was recently evaluated for cardiac cell transplantation, where magnetically labeled endothelial progenitor cells were injected in the infarcted myocardium while a magnet was externally applied to rats in the heart zone. Magnetically assisted cell delivery resulted in an increased concentration of cells and the short-term effect on cell retention was monitored *in vivo *by MRI and quantified by RT-PCR [42]. In a study evaluating the long-term engraftment, the functional benefits of magnetically assisted cell retention were also confirmed [43], improving cardiac ventricular function.

### Practical aspects for magnetic vectorization

**Figure 15:**
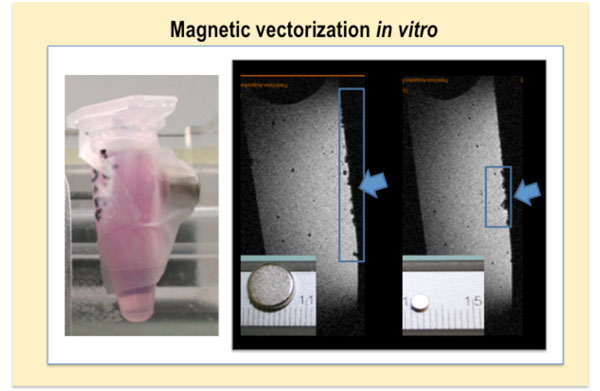
**Magnetic vectorization of cells in agarose gels with different magnets**. The figure shows the MR scans of two agarose gels, where magnets of different size and strength were put on the tube's surface (left panel). Blue squares and arrows indicate the zone where cells preferentially cumulate due to the applied magnet.

A first step towards an effective magnetic targeting is to prepare viable magnetic cells, which will be responsive to the given magnetic force. Before any *in vivo *application, we should therefore assess *in vitro *the best parameters for the magnetic manipulation of cells (Figure 15). Indeed the conditions in gels are only a poor approximation of what will happen after cell administration to living beings. Anyway, this pre-assessment does, however, allow us to see at least if we reached the minimal magnetization that might enable distal cell guiding. Before we translate cell vectorization to animals, we can also assess how magnet retains cells during the stirring of agarose phantoms. Other approaches might be more complex and can theoretically and experimentally evaluate the effect of cell aggregation after magnetic cell targeting in models simulating vessel bifurcation [44].

**Figure 16:**
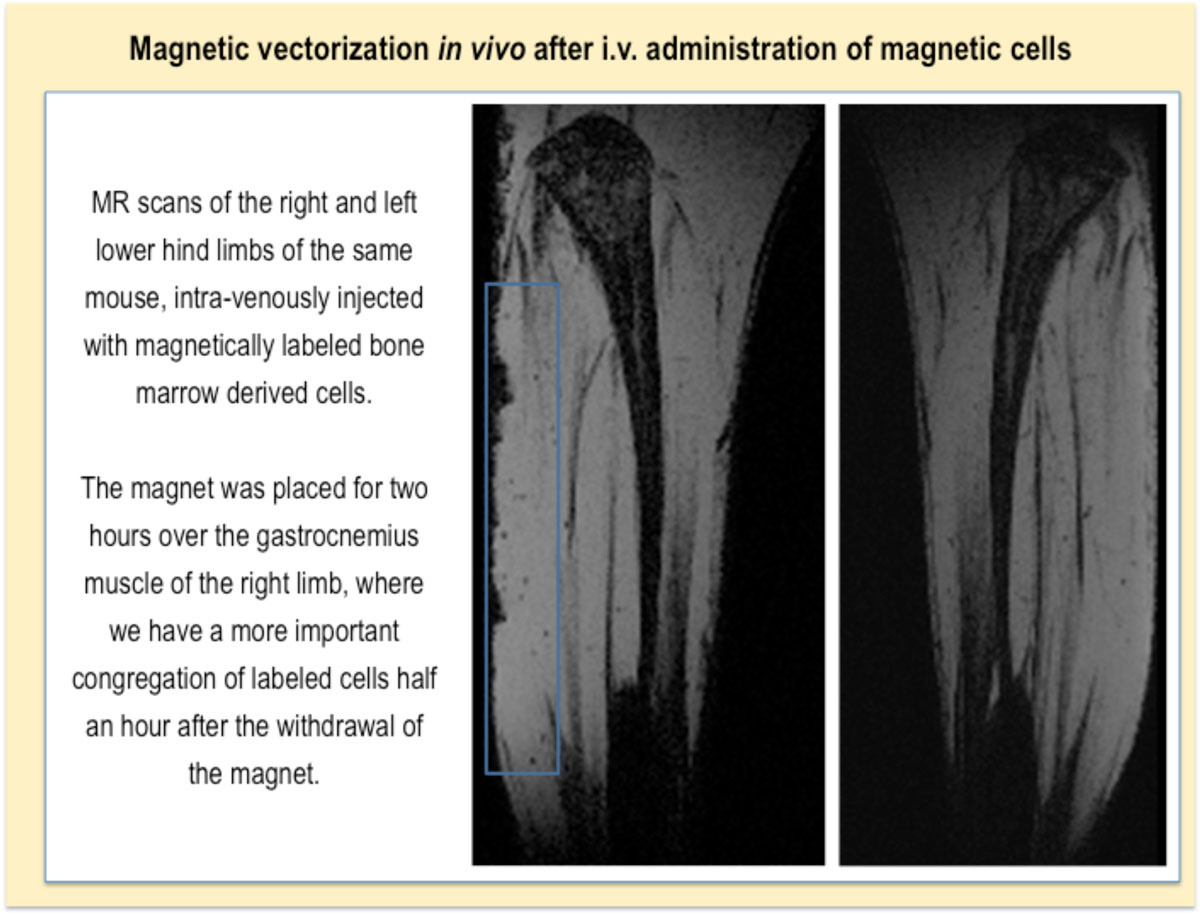
**Magnetic vectorization of labeled bone marrow derived cells in a healthy mouse**. MR scans showing hind limb scans of an animal. The blue square indicates the zone where cells preferentially cumulate due to the applied magnet.

Once we endowed cells with sufficient magnetization for cell vectorization, we can proceed to magnetic targeting *in vivo *(Figure 16). When we disperse labeled cells in the injection medium (which is generally the PBS), magnetic cells tend to be more prone to aggregation than non-labeled cells. If cell aggregates are injected intravenously, they will block the vessels (especially the small pulmonary ones) and the animals will die. In order to thoroughly disperse the cells, it is therefore necessary to disperse cells with a pipette cone or even pump the suspension in and out of the syringe for several times. This procedure might eventually lead to cell lysis, consequently, after such methods of dispersion, we should assess cell viability. If the viability is compromised, we have to disperse and administer cells by a syringe with a smaller gauge number (larger needle diameter). Nevertheless, even if administered cells are individualized, they might, under certain conditions, form perivascular aggregates within the body after magnetic guiding [45].

As we mentioned in the previous section, MRI allows *in vivo *follow up of magnetic cells and could be therefore used to confirm successful magnetic cell targeting. Nevertheless, in addition to this method, we should confirm that visualized spots correspond to injected cells. This might be done by immunohistological methods or flow cytometer analysis *post mortem*. Sometimes, especially if the cells are administered in low concentrations and systemically, the cells are difficult to find both by histology and flow cytometry. If we cannot localize the cells with these or other methods of cell detection, we should at least have a proof of an important therapeutic effect that could serve as a surrogate marker of cell delivery and local action.

## Summary and conclusion

Iron oxide nanoparticles can be used for magnetic labeling of different types of cells. The labeling of living cells allows a variety of biomedical applications ranging from cell manipulation to diagnostics and regenerative medicine. This tutorial provides the basic requirements for efficient cell labeling with anionic (citrate coated) iron oxide nanoparticles and includes sections on troubleshooting to prevent the occurrence of potential cell damage during the labeling procedure. In addition, as single cells can be monitored by high resolution MRI, we provide some appreciation of cellular MRI and present an abridged method for the quantification of punctual signal voids that are generated *in vitro *and *in vivo *by labeled cells. Finally, we also assess the potential of cell manipulation that can be exploited both in vitro for tissue engineering and *in vivo *in cell therapies.

## Competing interests

The authors declare that they have no competing interests.
